# Circadian phase response curves to light in older and young women and men

**DOI:** 10.1186/1740-3391-5-4

**Published:** 2007-07-10

**Authors:** Daniel F Kripke, Jeffrey A Elliott, Shawn D Youngstedt, Katharine M Rex

**Affiliations:** 1 Department of Psychiatry and Sam and Rose Stein Institute on Aging, University of California, San Diego #0667, La Jolla, California 92093-0667, USA; 2 Department of Exercise Science, Norman J. Arnold School of Public Health, University of South Carolina, Columbia, SC 29208, USA and Dorn VA Medical Center, Columbia, SC 29209, USA

## Abstract

**Background:**

The phase of a circadian rhythm reflects where the peak and the trough occur, for example, the peak and trough of performance within the 24 h. Light exposure can shift this phase. More extensive knowledge of the human circadian phase response to light is needed to guide light treatment for shiftworkers, air travelers, and people with circadian rhythm phase disorders. This study tested the hypotheses that older adults have absent or weaker phase-shift responses to light (3000 lux), and that women's responses might differ from those of men.

**Methods:**

After preliminary health screening and home actigraphic recording baselines, 50 young adults (ages 18–31 years) and 56 older adults (ages 59–75 years) remained in light-controlled laboratory surroundings for 4.7 to 5.6 days, while experiencing a 90-min ultra-short sleep-wake cycle. Following at least 30 h in-lab baseline, over the next 51 h, participants were given 3 treatments with 3000 lux white light, each treatment for 3 h, centered at one of 8 clock times. The circadian rhythms of urinary aMT6s (a melatonin metabolite), free cortisol, oral temperature, and wrist activity were assessed at baseline and after treatment.

**Results:**

Light (3000 lux for 3 h on 3 days) induced maximal phase shifts of about 3 h. Phase shifts did not differ significantly in amplitude among older and young groups or among women and men. At home and at baseline, compared to the young, the older adults were significantly phase-advanced in sleep, cortisol, and aMT6s onset, but not advanced in aMT6s acrophase or the temperature rhythm. The inflection from delays to advances was approximately 1.8 h earlier among older compared to young participants in reference to their aMT6s rhythm peaks, and it was earlier in clock time.

**Conclusion:**

In these experimental conditions, 3000 lux light could shift the phase of circadian rhythms to about the same extent among older and young adults, but the optimal light timing for phase shifting differed. For an interval near 4 PM, bright light produced only negligible phase shifts for either age group.

## Background

The phase of a circadian rhythm reflects where the peak and the trough occur, for example, the peak and trough of performance within the 24 h. It may be desirable to shift this phase, for example, to shift the time when peak performance occurs. Normally, circadian rhythms are synchronized with the 24.0 h environment by stimuli which alter the phase of the underlying brain circadian pacemaker. For most organisms, including mammals, the primary phase-shifting stimulus is light [1]. Effects of light in shifting human circadian rhythms have been described for several decades [2].

The relationship of the phase shifts in the organism to the circadian timing of the stimulus is called the phase response curve or PRC [3,4]. Several partial or complete PRCs of the human response to light have been experimentally determined [5-12].

More knowledge of human phase response curves to light is needed, now that bright light is being used increasingly to correct circadian rhythm phase disorders such as delayed sleep phase syndrome, to help air travelers adapt to jet lag, and to assist shift workers with adjustment to difficult schedules. The phase-shifting effects of light may also be relevant to treatment of depression [13-16]. The limitations of currently-available human PRC data have included a predominant focus on males (with very little phase-response data on women available), a paucity of comparative data by age groups contrasted simultaneously, and insufficient data points to accurately determine the shape of the PRC over the entire circadian cycle with stimuli of varying strength.

Different light stimuli patterns may produce PRCs of different shape and amplitude. Some previous PRC studies have used 5–6 h durations of extremely bright light (10,000 lux) which might be poorly tolerated or difficult to apply in normal life. In this study, 3000 lux (in a horizontal direction of gaze) was selected as approximately the brightest stimulus which could be practically and comfortably applied through overhead lighting in our isolation rooms. A 3-h stimulus was chosen to fit within the timing of our experimental model and be somewhat comparable to the exercise duration. The light stimuli were given on 3 consecutive days to augment the effect, as other studies have previously done [6]. Note that light stimuli on 3 consecutive days may produce entrainment responses qualitatively different from those of a single stimulus, because the circadian system may shift its phase during the interval while the light pulses are being given.

Because of concern that aging or gender might diminish phase-shifting responses, this research was planned to examine light PRCs simultaneously obtained from groups of women and men of both older (ages 59–75) and young-adult age groups (ages 18–31). The participants were randomly assigned to either bright light or treadmill exercise stimuli, so that light and exercise PRCs could be contrasted. The exercise PRCs will be reported elsewhere. This presentation will describe the light PRCs obtained from two different age groups and both genders.

## Methods

By advertising and word of mouth, the investigators recruited 337 volunteers who signed initial consent for the study. The study was approved and undergoes continuing annual review by the UCSD Human Research Protections Program (IRB) and the affiliated IRB of the VA San Diego Healthcare System. It was conducted in accord with the principles expressed in the Declaration of Helsinki. The target ages for young adults were 18–30 years and for older adults were 60–75 years. We sought both older and young-adult participants who were aerobically fit and in good general health, so that they would be capable of undergoing the exercise condition if randomized to that treatment. An inclusion criterion was regular participation in aerobic exercise for ≥20 min/day, ≥3 times/week at an intensity of ≥60% of maximal effort. Many of the volunteers were quite successful competitive endurance athletes (particularly those in the older group). All volunteers underwent medical histories, physical examinations, blood sugar, cholesterol and lipoprotein screening, and physician-supervised monitored exercise to verify the absence of EKG abnormalities. About 1/3 of the initial volunteers were dropped during the screening process for exercise safety considerations (e.g., high cholesterol or EKG abnormalities during monitored exercise) or because they decided they did not wish to complete the protocol. Also, potential participants were excluded if they took medications thought to influence melatonin or cortisol (e.g., melatonin, beta blockers, high doses of aspirin, corticosteroids). More older than young participants were recruited, because it was predicted a larger N might be needed for adequate power to detect a PRC in the older age group.

Preliminary screening studies included sleep and medical history forms and the Pittsburgh Sleep Quality Index (PSQI) [17]. Some of the PSQI results have been reported elsewhere [18]. For 7 days before entering the laboratory, participants wore the Actillume-I wrist actigraph for continuous 24-h recording of activity and illumination exposure. Sleep-wake was inferred by validated algorithms [19,20]. During the same week, participants completed home sleep logs estimating sleep time and quality, and completed a baseline Center for Epidemiologic Studies Depression Scale (CESD) [21]. The CESD was repeated both on the first and last days in the laboratory and one week later, to measure any mood effects of the interventions. Participants were asked to abstain from alcohol and caffeine for 2 days before entering the laboratory.

Participants first entered the Circadian Pacemaker Laboratory at about 09:30 and were assigned to individual studio apartments with sound and light isolation. They were asked to remain in their rooms or in a hallway with illumination limited to 50 lux for the duration of their time in the laboratory, from 4.7 to 5.6 days. They were not permitted in distant parts of the laboratory near windows or daylight. During their entire time in the laboratory, they were instructed to follow a special ultra-short sleep-wake cycle, consisting of 30 min in bed in complete darkness with sleep encouraged, followed by 60 min out of bed in background illumination, which was maintained at <50 lux in the usual direction of gaze. The ultra-short sleep wake cycle is a protocol used successfully by several laboratories to reduce sleep and light masking of circadian rhythms [22-26]. Although maintained in standardized lighting and ultra-short sleep-wake cycles, participants' social interactions were not restricted. Visitors and contacts with staff were permitted, along with reading, watching television (less than 10 lux), craft projects, working at computer games (less than 8 lux), telephone calls, and preparing meals. Strenuous exercise was not permitted.

**Figure 1:**
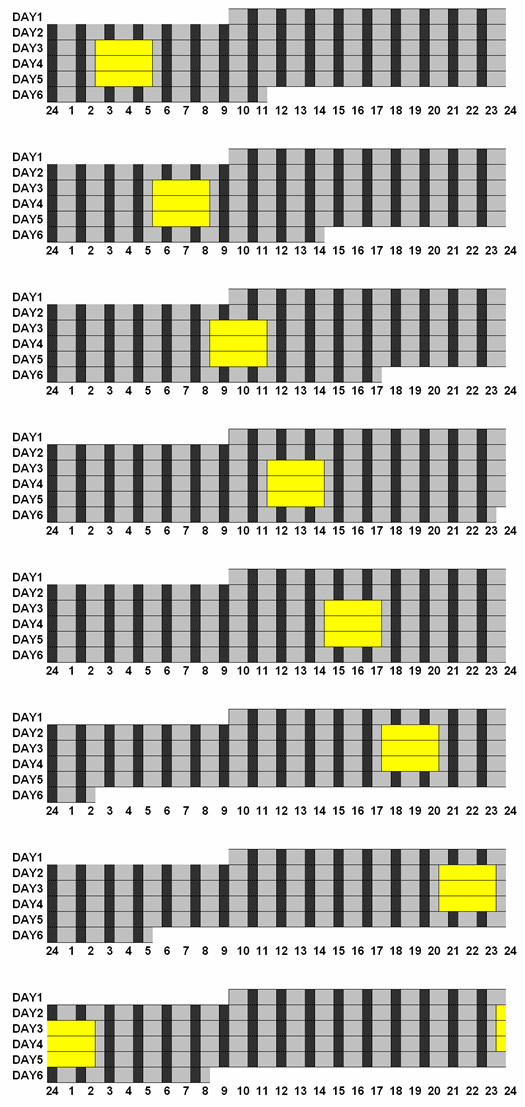
**The experimental protocols**. The experimental protocols are shown with an ordinate of 1 line per day and an abscissa of 24 h from midnight to midnight. Volunteers arrived in the laboratory at 09:30 on day 1. The ultra-short sleep-wake cycle, consisting of 30 min for sleep (black bars) followed by 60 min for wake (shaded bars) began at 10:30 and continued for 4.7 to 5.6 days. Three consecutive treatments (3 h bright light, yellow areas) were commenced after 38–54 h of baseline at one of 8 times. Circadian phase was assessed during the final 24 h of baseline preceding the first experimental treatment and for 24 h starting 6 h after the last treatment.

**Figure 2:**
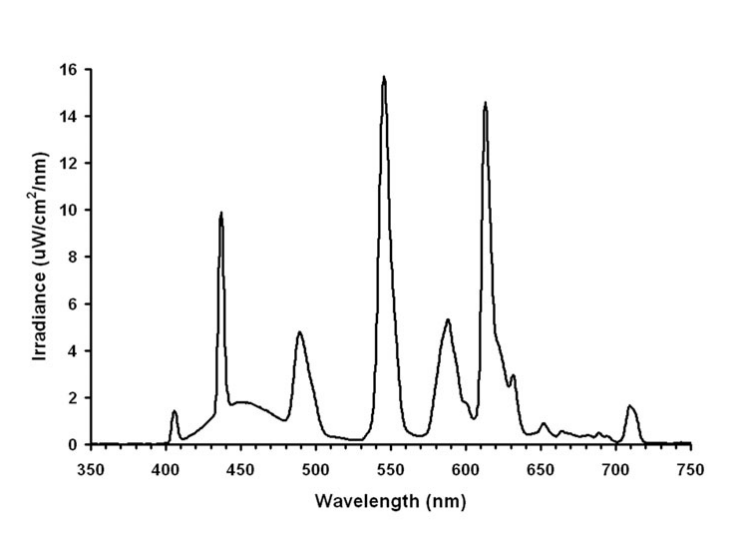
**The light spectrum of the bright light treatment**. The spectral content of the bright light treatment measured at eye level (standing) was averaged for the 3 subject rooms. The abscissa is wavelength in nanometers.

Baseline observations were continued for the first 30–53 h, of which the final 24 h were analyzed for baseline circadian assessments. Almost all participants were randomized to receive bright 3000 lux light stimuli or exercise when they first entered the laboratory, without being advised in advance of what treatment they would experience at what times of day. Because of difficulties recruiting healthy participants, 7 older volunteers were invited to enter the light protocol several months after having completed the exercise protocol to which they had initially been randomized. After a baseline of varying length, participants commenced bright light exposures centered at one of 8 times: 0100, 0400, 0700, 1000, 1300, 1600, 1900, or 2200 h. The 8 protocols are illustrated in Fig. 1. A 3-h block of bright light treatment was administered at the same time of day for 3 days. The bedrooms of about 18 m^2 ^were painted with white reflective paint. The ceilings had 8 recessed fixtures, each with a diffuser covering six 4-foot T12 cool white 4100, 40-watt fluorescent bulbs (Philips F4C Advantage X). The lights were controlled externally. For bright light treatments, all bulbs were lit, whereas for 50 lux, only one dimmer bulb was used. The ceiling fluorescent lighting provided approximately 3000 photopic lux to the cornea in a horizontal direction of gaze (see Fig. 2). Structured block randomization was employed so that approximately equal numbers were assigned to each of the 8 bright light stimulus times.

Participants continued to wear the Actillume wrist actigraphs throughout their time in the laboratory. Oral temperatures were taken with fast-reacting high-resolution electronic thermometers every 30 minutes. Because of superior circadian goodness of fit, only those oral temperature measurements obtained every 90 min immediately after awakening were used to obtain circadian analyses. Since these latter temperature measurements were each made in bed after 30 min. lying in bed, temperature was measured in a sort of constant routine which would minimize any effects of posture, activity, or meals. We did not think that the advantages of rectal temperature recording would outweigh the inconvenience and risks to participants. During the baseline and again after the final bright 3000 lux light stimulus, every urine voiding was collected. With few exceptions, participants provided a urine specimen each 90 min during lights-on, drinking at least 200 cc every 90 min to maintain steady production. The volume of each urine sample was measured and aliquots (2 ml) were immediately frozen and then soon transferred to -70°C, where the samples were stored for later assays of 6-sulphatoxymelatonin (aMT6s) and urinary free cortisol. Visual-analog 100 mm line ratings were given on 8 scales every 3 h: these scales were ALERT, SAD, TENSE, EFFORT, HAPPY, WEARY, CALM, SLEEPY, AND OVERALL. Monk and colleagues have validated similar scales in time-isolation laboratory settings [27]. The CESD inventory was repeated near the beginning and towards the end of the laboratory stay.

### aMT6s

The aMT6s assays were performed using Bühlmann 96 well ELISA kits (EK-M6S) purchased from ALPCO, Ltd. (Windham, NH). At the usual dilution of 1:200, the analytical sensitivity of the EIA was 0.35 ng/ml and the functional least detectable dose was 1.3 ng/ml for coefficients of variation (CVs) <20%. In our laboratory, control urine samples averaging 4–6 ng/ml gave intra- and inter-assay CVs of 4% and 7%, respectively. All samples from an individual participant were run at the same time and wherever possible on the same 96-well plate. Selected samples (especially peak or "circadian night" samples measuring > 38 ng/ml or samples < 1 ng/ml) were assayed repeatedly at either increased (1:800 to 1:3200) or decreased (1:25 to 1:100) dilution when necessary to obtain more accurate estimates or to clarify irregular circadian patterns in excretion rate (ng/hr).

From the aMT6s concentration, the urine volume, and the collection times, the aMT6s excretion rate (ng/h) was computed for each collection interval (the interval between one voiding and the next one) and subsequently associated with each 5-min interval within the collection interval. From this time series of 5-min intervals, the circadian analyses were computed (see below).

### Urinary free cortisol

Urine samples were assayed for free cortisol using DSL-2100 Active Cortisol RIA kits (Diagnostic Systems Laboratories, Inc. Webster, Texas). Because our 90 min sampling protocol typically yielded somewhat dilute urine, the urine sample volume in the RIA was increased to 75 μl combined with 25 μl of zero calibrator, adjusting the volume of kit standards and controls accordingly (e.g. 25 μl standards plus 75 μl deionized water). A low dose control (mean 1.3 μg/dL) run in triplicate in 12 assays gave intra- and inter-assay coefficients of variation (CVs) of 6.8% and 8.7%, respectively. Samples measuring <0.16 or >20.0 μg/dL when run at 75 μl were reassayed using either 250 μl or 25 μl of sample to obtain more accurate estimates. As with aMT6s, the cortisol concentrations were used to infer cortisol excretion for each 5 min interval. Because the urine integrates the pulsatile secretion of cortisol into blood, fewer urine samples than blood samples are needed to obtain a precise assessment of the phase of the circadian system. However, interim analyses suggested that urinary cortisol was not yielding more reliable circadian information than aMT6s, so cortisol was not assayed for the final third of laboratory studies.

### Circadian Analyses

Separate analyses were done for the last 24 h of baseline 90-min sleep-wake cycle, before light treatment, and for the comparable final 24 h of follow-up laboratory 90-min cycle (starting 6 h after the end of light treatment to minimize transients). For measures such as urinary aMT6s, urinary cortisol, oral temperature, and actigraphic minute-by-minute scored sleep, the best-fitting 24-h cosine was estimated with a least-squares technique. Then the acrophases (peak of the fitted curve) and mesors (mean of the fitted curve) were obtained as the estimates of the daily mean excretion and circadian timing. Baseline results from some of the first participants in this study, combined with some of the participants who would be assigned to exercise, have been reported previously [18,28]. Each phase response resulting from bright light stimuli was then computed, e.g., as the acrophase of the baseline minus the acrophase of the follow-up interval. A negative phase shift indicated a delay, e.g., that the acrophase of the rhythm occurred at a later clock time after the stimulus than during baseline. A positive phase shift would indicate an advance, e.g., that the acrophase was at an earlier time at follow-up than at baseline. The phase shifts from baseline to follow-up were then related to the time lag between the center time of the 3-h light stimulus and the baseline acrophase of aMT6s (or temperature, cortisol, etc.) to form the phase-response curves for each variable studied with each phase reference.

At the same time that these experiments were performed, a separate group of men and women of similar ages were exposed to the 90-min ultra-short sleep-wake cycle in the same laboratory with no more than 50 lux light exposures [29]. These subjects appeared to free-run with a period averaging 24.38 h [29]. Thus, for the participants exposed to bright light, the phase shifts were interpreted as relative advances or delays in reference to their mean phase shift, which approximated the free-running delay among the untreated subjects. The mean of the participants undergoing bright light stimuli was regarded as the best estimate for their free-running trend, because the untreated subjects were selected by different criteria and were in the laboratory for a shorter duration, so their estimated free-running period might have been more affected by transients.

**Figure 3:**
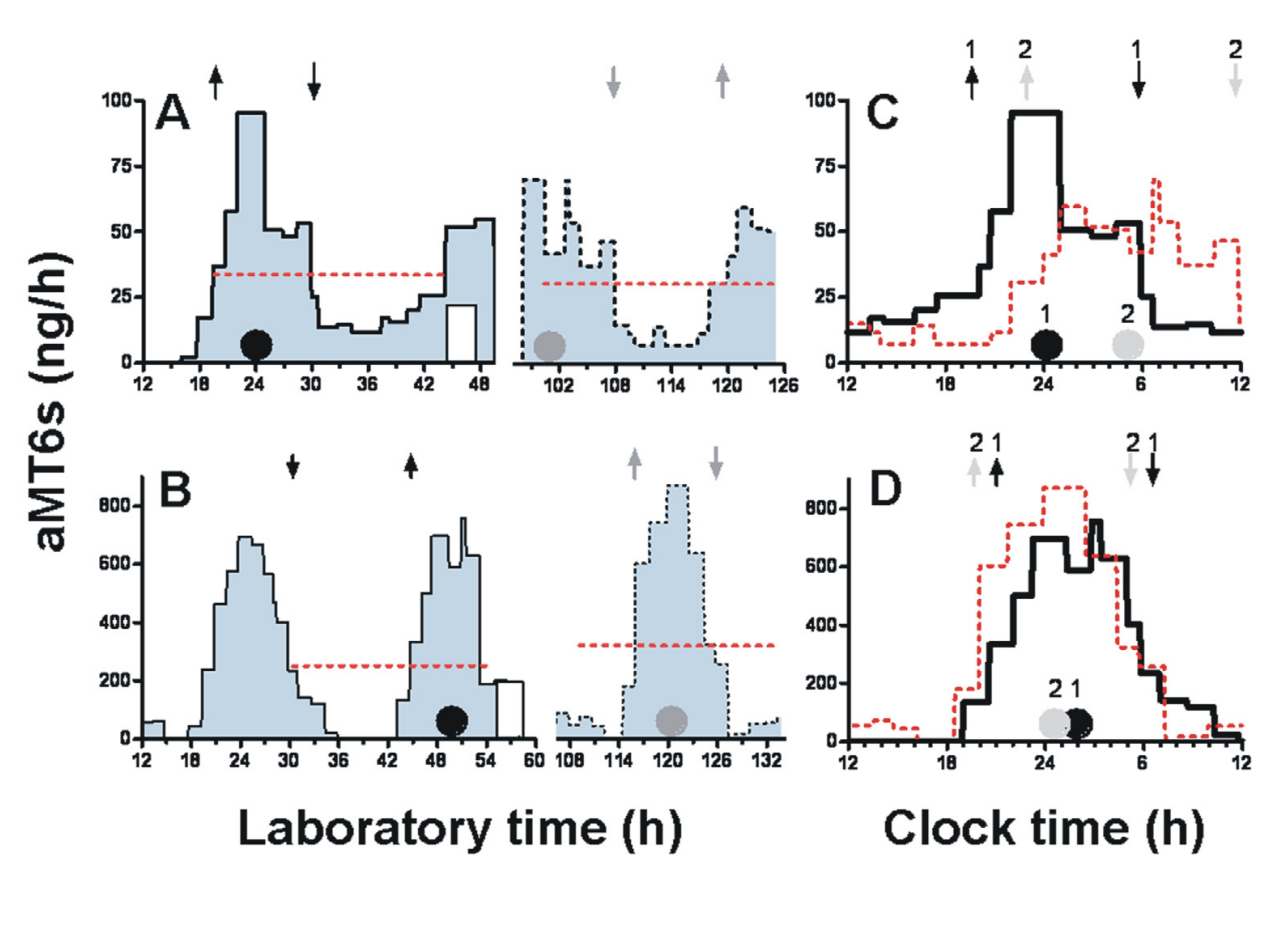
**Profiles of aMT6s excretion**. Examples of urinary aMT6s interpretation are plotted for one male participant, age 61 years (**A **and **C**), and one female participant, age 28 years (**B **and **D**). Panels **A **and **B **plot aMT6s (ng/h) in blue longitudinally during the two segments of continuous collection used for baseline and post-treatment phase assessment. The abscissa is h from the midnight commencing the first laboratory day (broken axis to omit 2 days of treatment). A cosine curve was fit to the 24 h immediately prior to the first light pulse (white bar) and again to the last 24 h. The horizontal red dotted lines represent the mesors (fitted means) associated with each cosine. Filled circles show the time of the cosine acrophases before (black), and after (grey) light treatment. Times of aMT6s onsets and offsets are represented respectively by upward and downward pointing arrows (black arrows for baseline and grey arrows for post-treatment.) The light-induced phase shifts in circadian aMT6s profiles are illustrated in panels **C **and **D **by replotting both baseline (1, black line) and post-stimulus curves (2, red line) on a noon-to-noon abscissa. In **A **and **C**, light given 8–11 PM, elicited phase delays of -5.0, -3.3, and -5.9 h, respectively, in the aMT6s acrophase, onset and offset. In **B **and **D**, the light stimulus given 5–8 AM produced phase advances of 1.2, 1.1, and 1.1 h, respectively. Note that the phase shifts were well-demonstrated despite the lower aMT6s excretion in the older participant.

**Figure 4:**
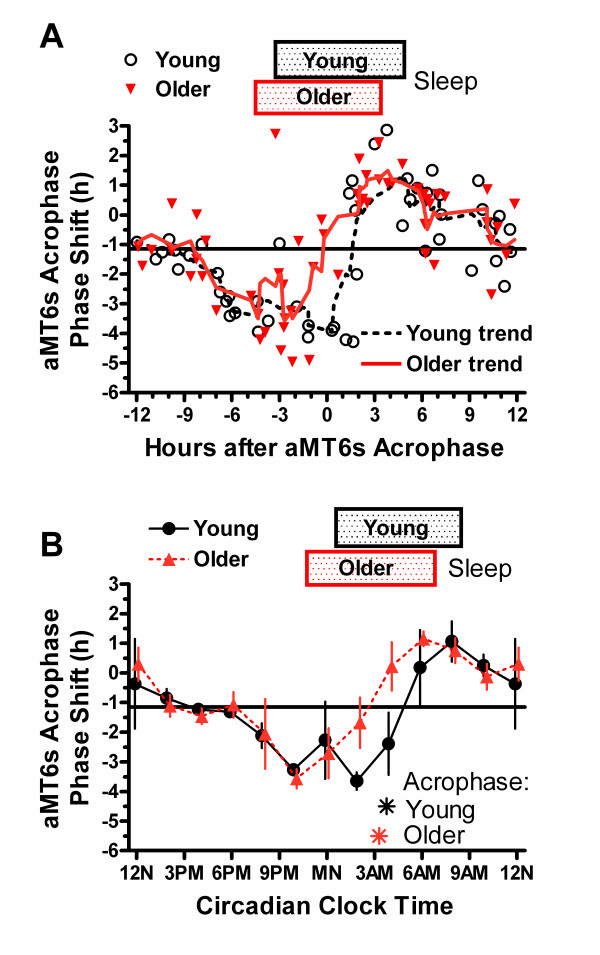
**Phase shifts of acrophase of aMT6s rhythm**. **A**. Phase shifts in the aMT6s circadian rhythm resulting from light stimuli are shown for 106 participants. The ordinate shows the shift in h of the aMT6s acrophase, computed as the baseline aMT6s acrophase minus the acrophase after the 3 bright light treatments. Thus negative shifts indicate that the follow-up acrophase was later than the baseline acrophase, i.e., delayed in clock time. The abscissa represents the timing of the midpoints of the 3-h light stimuli, as referenced to the baseline aMT6s acrophase. Stimuli given with an abscissa near 0 were approximately centered at the baseline aMT6s acrophase. Black circles represent phase shifts of individual young adult participants, and red triangles represent phase shifts of older participants. The solid black horizontal line shows the mean of all points, approximating the phase shift resulting from the circadian free-running component. The black dashed and red lines represent the trends from 5-point moving averages for the young and older groups. Rectangles illustrate the average actigraphic home sleep times for the young and older groups, referenced to their aMT6s acrophases. **B**. The phase shifts in aMT6s acrophase were averaged to show the mean ± 1 SEM for 2-h bins of time-of-stimulation referenced to the aMT6s acrophase. "The abscissa (Circadian Clock Time) references the midpoint of 3 h light stimuli to the time of the baseline aMT6s acrophase, and then displays the environmental time scale corresponding to when the mean aMT6s acrophase occurred at baseline. Thus the Circadian Clock Time abscissa (Figs 4-6) also represents our best estimate of the mean environmental clock time at which bright light stimuli occurred, adjusted for each participant's baseline circadian phase (aMT6s acrophase or onset). The asterisks illustrate the mean aMT6s acrophase times for the young and older groups.

**Figure 5:**
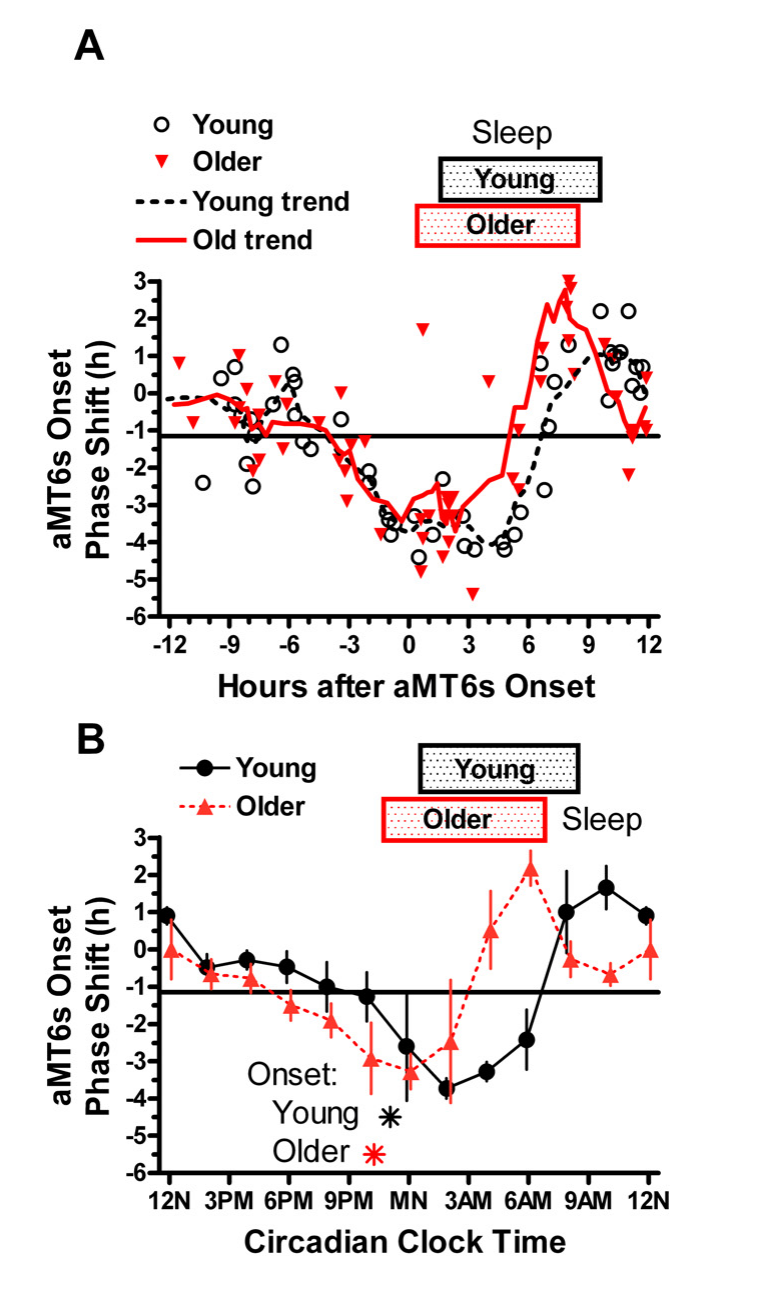
**Phase shifts of onsets of aMT6s rhythm**. **A**. Time shifts in aMT6s onsets are contrasted in young and older groups. The abscissa represents the time of bright light stimuli (midpoint of 3 h pulse) in reference to the time of the aMT6s onset at baseline. Trend lines for each age group represent 5-point moving averages. Relative to baseline aMT6s onsets, the inflection from delays to advances occurred earlier in the older adults. **B**. The shifts in aMT6s onsets were averaged to show the mean ± 1 SEM shift in onset times for non-overlapping bins of time-of-stimulation referenced to aMT6s onset. The abscissa (Circadian Clock Time) is our best estimate of the mean clock time at which bright light stimuli occurred, adjusted for each participant's aMT6s onset time. Asterisks represent the mean onsets for young and older groups.

**Figure 6:**
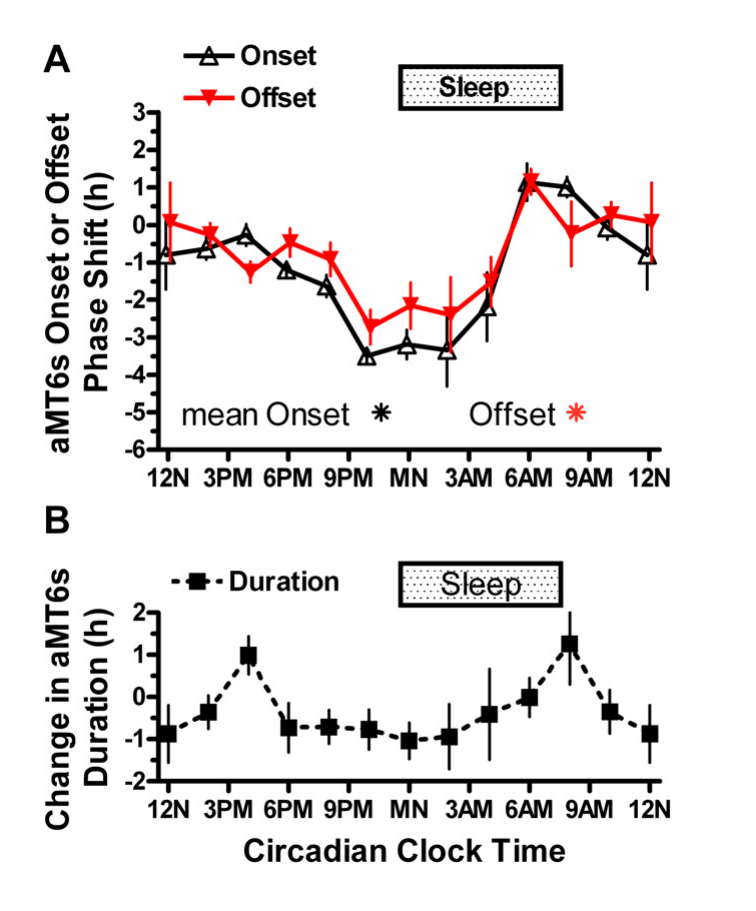
**Phase shifts of onsets, offsets, and duration of aMT6s rhythm**. **A**. The time shifts for aMT6s onsets (black triangles) and offsets (red triangles) are contrasted, after averaging the data in 2 h bins showing the mean ± 1 SEM shifts in time, referencing time-of-stimulation to aMT6s onset. The abscissa (Circadian Clock Time) represents our best estimate of the mean clock time at which bright light stimuli occurred, adjusted for each participant's aMT6s onset time, as in Figure 5. The asterisks show mean aMT6s onset and offset times for all 106 participants. **B**. The change in mean aMT6s duration resulting from unequal shifts in aMT6s onset and offset is plotted, using the same bins and abscissa as above.

To further describe changes in circadian phase and waveform, we estimated the circadian timing of nocturnal aMT6s onsets and offsets algebraically from upward (onset) and downward (offset) crossings of the mesor (ng/h), calculated from 24 h cosine fits to the data (Fig. 3). Shifts in onset and offset times were also computed. To aide interpretation of the PRC data in relation to clock timing in the home environment, some of the figures plotted phase shifts to light on a 24 h abscissa titled Circadian Clock Time (Figures 4, 5, 6). The abscissa Circadian Clock Time references the timing of light stimulation to a phase marker (i.e., aMT6s acrophase or onset), and then displays the environmental time scale corresponding to when the mean phase marker occurred at baseline. The mean phase markers used are also located on the time scale as asterisks. This form of display illustrates our best estimate of the mean environmental clock time at which the stimuli were given, adjusted for variations in each participant's baseline phase.

To test the null hypothesis that there were no phase-response curves, that is, no phase-shifts dependent on the timing of the 3000 lux light stimuli, we used both the PRC bisection test [30] and factorial ANOVA. The PRC bisection test locates the best bisection of the circular distribution of initial phases to maximize the contrast between advances and delays. In general, the best bisection will be at the inflection from delays to advances. The test then determines if the bisection separates advances and delays significantly better than would occur in a random distribution. These tests were performed for all 106 participants and on subgroups of older and young adults, male and female. The inflection points of the PRCs from delay to advance were estimated with the PRC bisection analyses. The amplitudes of PRCs were contrasted between older and young adult groups, men and women, using methods derived from the PRC bisections [30]. Because the PRC bisection test was a new approach, these tests were confirmed by factorial ANOVA, allocating the phase shifts into 6 prospectively-planned 4 h treatment-timing blocks (referenced to the baseline acrophases), and adding age group and gender as additional factors to produce 6 × 2 × 2 analyses. A criterion for significance of p < 0.05 was selected. No correction for multiple testing seemed appropriate, since most tests were significant, and correction would be problematic with tests which were intercorrelated.

## Results

A total of 50 young adults ages 18–31 years (mean 23) and 56 older adults ages 59–75 years (mean 67) completed the protocol and supplied usable data. The young adults included 31 women and 19 men. The older adults included 28 women and 28 men. Seven additional participants entered the laboratory, but dropped out in the first 2 days before receiving randomized treatment, mostly with headache complaints. These complaints decreased after a room ventilation problem was identified and resolved. One participant quit during bright light treatment because a personal responsibility arose unexpectedly. The studies were done from September, 1999 through March, 2003, both age groups and both genders being studied simultaneously at all times of year.

Some characteristics of the participants at baseline are shown in Table 1.

**Table 1:** Baseline Characteristics of Participants (Mean ± SD)

	**YOUNG ADULT**	**OLDER**	**AGE CONTRAST**
**Wake-up (questionnaire)**	7:31 ± 1:24	5:55 ± 1:18	P < 0.001
**Actigraphic wake time**	8:32 ± 2:16	6:39 ± 1:07	P < 0.001
**Bedtime (questionnaire)**	23:48 ± 1:03	22:51 ± 1:05	P < 0.001
**Bedtime (actigraph)**	00:33 ± 1:31	22:41 ± 1:24	P < 0.001
**Sleep log total sleep time**	444 ± 65 min	406 ± 63 min	P = 0.003
**Actigraphic total sleep time**	404 ± 70 min	385 ± 68 min	NS
**aMT6s onset**	23:04 ± 1:45	22:16 ± 1:46	P = 0.02
**aMT6s acrophase**	3:47 ± 1:37	3:18 ± 1:58	NS
**aMT6s offset**	08:22 ± 1:28	08:20 ± 2:11	NS
**Cortisol acrophase**	9:59 ± 1:54	8:29 ± 2:51	P = 0.008
**Oral Temperature bathyphase**	04:04 ± 2:10	04:44 ± 3:52	NS
**PSQI total score**	3.8 ± 2.3	3.6 ± 2.7	NS
**CESD at intake**	7.0 ± 7.0	3.5 ± 3.6	P = 0.002

NS = Not Significant. Temperature bathyphase: the fitted minimum, 180° from the acrophase.

The bright-light phase response curves for young and older adults are shown in Fig. 4, using the aMT6s acrophases as the reference. The participants showed a trend to delay an average of 1.09 h between the phase assessments, which were centered 81 h apart. This would correspond to a free-running tau of 24.32 h. There was no significant difference between the average delay of older and young adults, 0.93 and 1.27 h respectively. Light stimuli centered from 8 h before the aMT6s acrophase roughly up to the acrophase produced phase delays as referenced to this mean shift. Light stimuli in approximately the first 10 hs after the aMT6s acrophase produced phase advances as referenced to the mean shift. For the older participants, the inflection of the trend line from delays to advances crossed the mean phase response line about 0.2 h before the aMT6s acrophase, whereas among the young, this inflection crossed the mean phase response line at 1.6 h after the aMT6s acrophase, a difference of 1.8 h. Fig. 4B emphasizes that the PRC inflection times of the older subjects were more than 1.8 h earlier than those of the young in reference to clock time. From about 10 to 16 h after the aMT6s acrophase, the phase responses approximated the mean shift, averaging -1.06 h; that is, from 10 to 16 h after the aMT6s acrophase, there appeared to be a dead-zone region, during which no significant linear trend in the phase responses was observed.

Outcomes of the PRC bisection tests are summarized in Table 2, which gives the estimated angle of the inflection from the phase reference, the mean clock time to which that estimated inflection would correspond, the D parameter which reflected the amplitude of the PRC, the number of subjects, and the probability of the null hypothesis. Referencing phase responses in the aMT6s acrophases to the baseline aMT6s acrophases, the overall PRC bisection test for all participants gave a highly significant rejection of the null hypothesis of no PRC. This was also the case for subgroups of all older, all young, all males, and all females. The PRCs of each of these four subgroups were highly significant, had a similar D score estimating the amplitude of the PRC, and similar estimated phases of the inflection point from delays to advances (adjusted for mean shifts). The results for the much smaller age-gender subgroups shown in Table 2 were also rather similar, though the bisection test for older males missed the 0.05 significance criterion. Using factorial ANOVA to contrast PRC amplitudes derived from the PRC bisection procedure (see [30]), no significant differences in PRC amplitude were found between older and young groups or between female and male groups, nor was the age X gender interaction significant. It appeared that the largest difference in inflection phases was between older and young females, but the 95% confidence limits for inflection phases derived from the PRC bisection test overlapped when comparing the older and young female subgroups.

**Table 2:** PRC Bisection Test Inflection Phases and Clock Times of Inflection

**GROUP**	**INFLECTION referenced to acrophase**	**TIME (mean)**	**D**	**N**	**P**
aMT6s phase shifts referenced to aMT6s acrophase					

All ages	21.3°	4:56	36.9	106	<0.0001
Young males	21.3°	5:29	37.4	19	0.004
Young females	45.3°	6:37	38.7	31	0.007
Older males	29.1°	5:23	28.8	28	0.087
Older females	1.6°	3:49	45.35	28	<0.0001

cortisol phase shifts referenced to baseline aMT6s acrophase					

All ages	4.5°	3:49	35.6	73	0.0005

oral temperature phase shifts referenced to baseline oral temperature acrophase					

All ages	159.6°	3:31	55.0	96	<0.0001

lab actigraphic sleep phase shifts referenced to baseline aMT6s acrophase					

All ages	10.7°	4:14	38.0	102	0.03

Shifts in aMT6s onset, offset, and duration, with treatment time referenced to aMT6s acrophase					

aMT6s onset	27.5°	05:21	33.9	104	<0.0001
aMT6s offset	14.1°	04:28	22.5	104	<0.02
aMT6s duration*	29.1°	05:28	1.06 h	104	P < 0.01

* The bisection test was modified to determine if changes in aMT6s duration were random in phase

Whether the treatment time was referenced to the baseline aMT6s acrophase or to the baseline cortisol acrophase, cortisol phase responses were similar to those for aMT6s, but the bisection tests for cortisol were not significant for the younger group with N = 42. The D scores for oral temperature phase responses were rather high, but the bisection tests were not significant for the older participants, whether treatment timing was referenced to oral temperature baseline phases or to aMT6s baseline phases. Bisection results for actigraphic sleep phase responses resembled those of the other variables, but the tests were not as highly significant, in as much as the circadian amplitude/mesor ratios of actigraphic sleep rhythms were not as high as for the urine variables or oral temperature, and the circadian phase estimates were accordingly less precise.

Factorial ANOVA of aMT6s acrophase responses for all participants demonstrated a highly significant time-of-treatment effect (F_5,82 _= 15.98, P < 0.0001), confirming a significant PRC. There were no significant main effects of age group or gender and no interactions of age or gender with the time-of-treatment. However, when examining the phase shifts for the onset of aMT6s, referencing light treatment time to aMT6s onset, the interaction effect of age and time of treatment was almost significant (P = 0.054), reflecting the earlier inflection among older participants. A similar ANOVA for cortisol phase response showed no significant time-of-treatment effect, whether the time of treatment was referenced to baseline aMT6s or to cortisol acrophases. The time-of-treatment effect in ANOVA for temperature responses referenced to aMT6s was significant at the P = 0.01 level with no significant age or gender effects or interactions. Referenced to baseline temperature acrophases, this temperature ANOVA was slightly less significant, P = 0.023. In the sleep phase responses ANOVA, the time-of-treatment effect was barely significant with P < 0.05, but a time-of-treatment X gender interaction was significant with P = 0.007, and an age X gender interaction was significant with P = 0.005, without any interaction of age with time of treatment.

There was no time-of-treatment effect on the final aMT6s mesors or circadian amplitudes determined by cosine fits. Phase shifts in aMT6s onsets and offsets were generally similar to those of the acrophases. However, because the aMT6s onsets were more advanced in the older compared to younger participants than were the baseline acrophases, the entire PRC waveform for shifts in the older group was more clearly phase advanced when referenced to onsets in circadian-adjusted time (Fig. 5B). Moreover, because the aMT6s onsets delayed more than the offsets in the phase-delay regions of the PRCs, the post-treatment duration of aMT6s excretion was significantly related to time-of-treatment by the bisection test (P < 0.01), as shown in Table 2 and Fig. 6B. This effect on duration was confirmed by ANOVA (P < 0.05). Fig. 6B indicates that light stimuli given near 4 PM or near 8 AM induced a lengthening of the aMT6s duration.

In 41 pairings where PRC bisection tests and time-of-treatment ANOVA were both computed, the PRC bisection test yielded more significant P values in 27 cases and ANOVA in 14. The correlation of log-transformed P values for the two methods was r = 0.83.

The mean CESD score increased from 4.9 at home baseline to 6.5 on the first day in the laboratory, 7.6 on the last day in the laboratory, and 6.1 on one-week follow-up (p < 0.03, one-way ANOVA), indicating slight increases in depression from baseline. The CESD scores following bright light treatment were not influenced by the timing of the treatment. Examining visual-analog scores at the end of treatment (Day 5 average) with repeated measures ANCOVA, the time of day of treatment referenced to aMT6s acrophase produced no significant effects on mood. However, as the study terminated, compared to the young adults, the older participants rated themselves as significantly more alert, calm, happy, and better in the overall score, but less sleepy, tense, weary, and sad.

No serious or lasting adverse effects of these experiments were observed.

## Discussion

These results again demonstrate the circadian phase-shifting effects of bright 3000 lux light and describe phase response curves. These observations confirm previous results that the inflection time from delays to advances averages an h or two after midsleep, which tends to occur earlier in older adults. The inflection time in aMT6s phase shifts averaged slightly after the aMT6s acrophase. The phase shift inflections were within the confidence limits of the oral temperature bathyphases, which are the fitted temperature minima, 180 degrees after the acrophases. This would be consistent with the previous literature, which located the inflection at approximately the core temperature minimum. The study was prospectively designed to have adequate power to detect PRC's in groups of 48 participants or more. The highly significant results for the aMT6s PRCs for older and young, men and women, indicated that the study was adequately powered for this purpose.

An important finding was that the light phase response (PRC amplitude) in older participants was of similar size to that of young adults. No predicted reduction in phase response to light stimuli was observed among older participants. Sufficient data had not been available to prospectively predict the power of the model to contrast older and young adults, as power predictions for PRC amplitude are complicated by age differences in timing and wave form. In retrospect, the data indicated that the experiment had 80% power to detect if the PRC for one age had twice the amplitude as that for the other age group (with 0.05 significance, two-tailed). Thus, a small difference in the phase responsiveness between older and young participants could not be excluded. Similarities in delay responses to light between young and elder participants were previously reported by others [11,31]. In one of 4 statistical tests, a greater advance response was observed among young participants (P < 0.05 uncorrected for multiple comparisons), but interpretation was complicated by lack of gender-matching of the groups and a shift in the schedule for darkness and sleep [30]. One study suggested that older adults were equally responsive to light stimuli of the intensity we used, but older adults were less responsive to dimmer light stimuli [32]. Because that was a retrospective finding based mainly on 3 subjects, and the groups were not studied simultaneously, more study of responsiveness of aging adults to moderate light stimuli is needed. Since we have found that subjects exposed to more light at home are more advanced, decreased light responsiveness would not explain why older adults are more advanced [33]. It should be recognized that these older participants with a mean age of 67 were very healthy and aerobically fit. Their average aerobic capacity was in the top 10% for their age. Selection might have biased against older adults with visual handicaps, although participants did not undergo specific ophthalmologic screening. It remains plausible that an even older age group with substantial cataract or glaucoma might experience decreased light responses, partly because their handicaps might promote bright light avoidance [34]. One might not expect macular degeneration to have much effect on the retinal photic receptors which supply the retino-hypothalamic tract [35], but associated photophobia might limit daylight exposures.

These data allowed a contrast of light phase responsiveness between females and males of diverse ages. No significant difference between the phase responses of females and males was observed, though the statistical power was insufficient to detect small gender differences. Although D scores in Table 2 suggested that older females tended to be somewhat more light-responsive than older males, the difference was in the consistency of the PRC responses rather than in their range. It seems unlikely that light phase responsiveness accounts for differences in home circadian phase adjustments between females and males.

An unusual feature of our study was the computation of PRCs in multiple dependent variables, using acrophases of multiple variables to reference the timing of bright light treatments. As we had anticipated, aMT6s provided the most reliable marker that produced the most consistent responses, as judged by the PRC amplitudes and statistical significance. The PRCs using oral temperature, urinary free cortisol, and actigraphic sleep as phase markers for the time of light stimulation were consistent with the PRCs using aMT6s for reference. Likewise, the PRCs for temperature, cortisol, and sleep had similar D scores summarizing PRC amplitudes and similar estimated inflections. The temperature, cortisol and sleep PRCs for some subgroups were less statistically significant, probably because of less reliable physiologic measurement, less sinusoidal circadian waveforms, and in some cases, less usable data available. For this reason, this presentation focuses on aMT6s data. Parenthetically, this was our first extensive utilization of the PRC bisection test, which was designed for this study. Although statistical significance as assessed by ANOVAs was well correlated with significance estimated by PRC bisection tests, in almost 2/3 of comparisons, the PRC bisection test result proved more significant, and the bisection test result was more often significant than ANOVA.

From a theoretical viewpoint, since melatonin causes much of the circadian variation in core body temperature [36] and is masked little by sleep, melatonin is a logical choice of circadian marker. Since the PRC's for the urinary metabolite of blood melatonin, aMT6s, were more consistently statistically significant than PRC's for oral temperatures or other circadian markers, but not of higher amplitude, it appeared that the acrophases of aMT6s had less measurement error. We found actigraphic sleep very difficult to score in the ultra-short sleep-wake cycle model, and apart from scoring problems, we would expect sleep-wake to be less tightly synchronized to the suprachiasmatic nucleus circadian pacemaker than melatonin or cortisol. To the extent that the sleep-wake circadian rhythms were of marginal amplitude, the 90-min sleep-wake cycle presumably reduced sleep masking of other variables. Cortisol and melatonin might be expected to be tightly locked in phase, since both circadian rhythms are controlled by suprachiasmatic nucleus efferents to the hypothalamic paraventricular nucleus, but for demonstrating a PRC, aMT6s appeared the more convenient and informative marker.

In these subjects, we found that sleep-wake at home and cortisol circadian rhythms in the lab baseline were significantly advanced among the older adults (ages 59–75) as compared to the young (ages 18–31), as was the aMT6s onset. In some of the same subjects, Yoon et al. found that the onset of melatonin excretion tended to be advanced along with sleep and cortisol, but the offset of melatonin was less advanced, resulting in a longer duration of estimated melatonin excretion among the older participants [28], as was likewise indicated in Table 2. This was largely consistent with findings of other groups [37-39]. Similar contrasts of young and older adults were also found in less healthy elders using the same 90-min-day protocol [29]. In the latter study, slowed metabolism of melatonin among older participants seemingly could be excluded, suggesting that it was the actual offset of synthesis of melatonin in the pineal, controlled by the suprachiasmatic nucleus circadian system, which became relatively delayed in reference to the sleep and cortisol rhythms. That body temperature was not found to be significantly advanced among older participants may be partly attributable to the regulation of body temperature by melatonin and partly a reflection of less accurate circadian temperature measurement. This might raise the question whether sleep-wake and cortisol assume a more advanced phase-angle versus the suprachiasmatic circadian pacemaker as we age, or whether body temperature and melatonin secretion become more delayed, at least in the melatonin synthesis offset.

Fig. 4 suggests that the PRC inflection from delay to advance occurred earlier in reference to the aMT6s acrophase among the older adults. It was also earlier in reference to clock time. Assuming that the PRC inflection is a good marker of the phase timing of the suprachiasmatic nucleus circadian oscillator, Fig. 4 implies that among older adults, the phase state of the suprachiasmatic nucleus circadian oscillator does become advanced, but the acrophase of the melatonin rhythm becomes relatively delayed in reference to that suprachiasmatic nucleus oscillator because the melatonin offset becomes delayed and melatonin duration grows longer. Perhaps because core temperature is governed in part by melatonin, it was not surprising that the temperature acrophase was also delayed in reference to the PRC inflection among the older participants. Although the graphs appeared to demonstrate a persuasive age difference in the inflection points, since the statistical evidence was marginal that the PRC inflection was earlier in reference to aMT6s acrophase among older participants, more study of this issue is needed. A possible explanation might be that when older adults arise early, sometimes before dawn, early morning illumination bright enough to advance or acutely suppress secretion of melatonin might not be experienced correspondingly early [29]. By this mechanism, aging-associated phase advances might influence the entrainment of the complex circadian oscillator in a manner somewhat analogous to the long nights of winter [40], which prolong the duration of the circadian subjective night. It is entrainment state which is at issue, since masking by light or sleep was equivalent among older and young adults during the laboratory baseline urine collections.

A delay in the offset of melatonin after awakening, when experienced for several weeks or more, has been suspected as a cause of depression [14,41]; however, no correlation was found among current participants between CESD in the laboratory and any lag of aMT6s offset after average home actigraphic wake time. A prolonged melatonin duration (prolonged subjective night) might tend to increase the phase responsiveness to light of elders, which could possibly compensate for reduced ophthalmic light transmission [42-44]. Fig. 6B shows that the specific timing of light stimuli varied the duration of melatonin secretion acutely, but further study is needed to learn whether this would occur with chronic treatment of a subject synchronized in the home environment.

In this experimental model, we observed a dead zone of approximately 6 h duration surrounding the core temperature acrophase, when little if any circadian phase shift as referenced to the mean shift was produced by 3000 lux light stimuli. This was in contrast to a previous report which observed no dead zone [45]; however, the trials in that study involved strong and complex stimuli, including shifting the times of 8-h intervals of bed rest in darkness. More moderate bright light stimuli produced human PRCs with a dead zone [8,46]. A relationship of PRC stimulus strength to the presence or length of a dead zone has been observed in laboratory animals. The existence of a dead zone in human PRCs is an important observation, indicating that most light exposures of the strength we employed would have minimal phase-shifting effects during much of the daytime h (e.g., from approximately 1:40 PM until 7:40 PM for a person awakening around 7 AM.)

The amplitude of the aMT6s PRC (Fig. 4, 5) was approximately 6 h, ranging from the maximal delay to the maximal advance in the averaged curves. If we assume that the mean acrophase shift of 1.09 h was attributable to a free-running component, 3 h was the maximal delay or advance phase shift produced by 3 h of 3000 lux light administered 24 h apart on 3 successive days. This was roughly consistent with previous studies [8,32,46]. Our PRC data as well as most other reports indicate that it would be quite difficult to quickly achieve the 8–12 h phase shifts desired by some air travelers and shiftworkers. Complex aspects of experimental design can influence the magnitude of resultant phase shifts and the shape of PRCs. Factors influencing PRCs include stimulus parameters such as the brightness and color spectrum of the stimulus, the duration of daily exposure, the number of repetitions (days) of stimulation, and the light levels experienced at other times of day as well as management of sleep, diet, posture, and social interactions. Because of photostasis, retinal adaptation to light may alter responses [47], so other factors might include illumination levels experienced before entry into the experiment, the duration and the intensity of baseline illumination. The duration of the prior photoperiod also may influence the shape and amplitude of the PRC [42-44]. Much more data are needed to clarify these many issues and to optimize phase responses.

## Conclusion

These experiments showed relatively similar amplitudes of phase response to 3000 lux light among older and young adults, and among women and men. The timing of the advance and delay regions of the older and young adults is clarified, under stimulation with moderately bright light. Among older adults, the PRC and its inflection time was substantially advanced in reference to clock time. Additionally, there was suggestive evidence for subtle rearrangement of the phase relationships between various phase markers among older adults, the implications of which deserve further exploration.

## Competing interests

The author(s) declare that they have no competing interests.

## Authors' contributions

DFK planned the study, was Principal Investigator of HL61280, supervised the staff, assured participant safety, contributed to statistical analyses, and wrote the first draft of the manuscript. JAE planned the study, performed the assays and endocrine analyses, prepared most of the figures, and contributed to writing. SDY planned the study, recruited and screened participants, supervised technicians and the day by day data collection, assembled the data base, and contributed to data analyses and writing. KMR took part in grant and study planning, handled administrative and budgetary aspects of the study, maintained human subjects protection files, supervised technicians, took part in data collection, and contributed to writing. All authors read and approved the final manuscript.
